# Acute Effect of Cigarette Smoking on Pupil Size and Ocular Aberrations: A Pre- and Postsmoking Study

**DOI:** 10.1155/2015/625470

**Published:** 2015-01-29

**Authors:** Uzeyir Erdem, Fatih C. Gundogan, Umut Aslı Dinc, Umit Yolcu, Abdullah Ilhan, Salih Altun

**Affiliations:** ^1^Ophthalmology, GATA Medical School, 06010 Ankara, Turkey; ^2^Ophthalmology, Yeditepe Eye Hospital, 34330 İstanbul, Turkey

## Abstract

*Aim.* To evaluate the acute effects of cigarette smoking on photopic and mesopic pupil sizes and wavefront aberrations. *Methods.* Cigarette smoker volunteers were recruited in the study. Photopic and mesopic pupil sizes and total ocular aberrations were measured before smoking and immediately after smoking. All volunteers were asked to smoke a single cigarette containing 1.0 mg nicotine. Pupil sizes and total ocular aberrations were assessed by optical path difference scanning system (OPD-Scan II ARK-10000, NIDEK). Only the right eyes were considered for statistical analysis. The changes of pupil size and total ocular aberrations after smoking were tested for significance by Wilcoxon signed ranks test. *Results.* Mean photopic pupil size decreased from 3.52 ± 0.73 mm to 3.29 ± 0.58 mm (*P* = 0.001) after smoking. Mean mesopic pupil size was also decreased from 6.42 ± 0.75 mm to 6.14 ± 0.75 mm after smoking (*P* = 0.001). There was a decrease in all the measured components of aberrations (total wavefront aberration, higher-order aberration, total coma, total trefoil, total tetrafoil, total spherical aberration and total higher-order aberration) after smoking; however the differences were insignificant for all (*P* > 0.05). *Conclusion.* Our results indicate that pupil constricts after smoking. On the other hand, smoking does not alter ocular aberrations.

## 1. Introduction

Nicotine is the primary active pharmacological agent in tobacco products, accounting for only acute pharmacological effects of smoking [1]. The other compounds of tobacco are responsible for the adverse long-term cardiovascular, pulmonary, and carcinogenic effects. Nicotine is a nonselective cholinergic nicotinic agonist that markedly affects various physiological parameters including heart rate, blood pressure, the electroencephalogram, and deep tendon reflexes, such as the patellar and Hoffmann reflexes [2–5].

Autonomic nervous system is composed of sympathetic and parasympathetic nervous systems. Sympathetic nervous system has adrenergic receptors at preganglionic and postganglionic terminals. Parasympathetic nervous system has cholinergic nicotinic receptors at somatic motor and preganglionic terminals and cholinergic muscarinic receptors at postganglionic terminals. It is known that iris receives dual innervation from both the sympathetic and the parasympathetic nervous systems, the sphincter iris muscle and the dilatator iris muscle being under the control of sympathetic and parasympathetic nerves, respectively [6, 7].

Wavefront aberration maps display the deviation of an emmetropic eye through the entire optical system and higher order aberrations are calculated from Zernike polynomials. Zernike polynomial coefficients of these higher order aberrations cannot be corrected with spectacles or lenses, and represent the magnitude of the group of wavefronts in units of microns [8]. The pupil diameter is known to be effective on the illuminance and sharpness of retinal image in a reciprocal manner; as the pupil dilates, the retinal image becomes more luminous, but wavefront aberrations tend to increase [9–12].

OPD-Scan II ARK-10000 is a refractive power analyzer, wavefront analyzer, corneal topographer, keratometer, autorefractor, and pupillometer all in one unit [13]. In this study we aimed to examine the acute effects of cigarette smoking on photopic pupil size, mesopic pupil size, and wavefront aberrations of the eye in a group of young male smokers using OPD-Scan system.

## 2. Methods

Regular cigarette smoker volunteers from the staff of our center were recruited in the study. The participants were required to meet the criteria of having smoked 10 or more cigarettes per day for at least five-year period. An informed consent was obtained from all of the patients, and all tenets of the Declaration of Helsinki were followed. Participants were healthy with no ocular abnormalities other than a refractive error.

All subjects were asked to abstain from tobacco smoking and caffeine containing products starting at 08.00 pm on the evening prior to the measurements until between 08:30 and 10:00 am. Smoking was performed using a single cigarette containing 1.0 mg nicotine (Pall Mall Filter, British American Tobacco). Photopic and mesopic pupil sizes, corneal topography, and wavefront measurements were assessed before smoking and immediately after smoking by optical path difference scanning system (OPD-Scan II ARK-10000, NIDEK).

The OPD-Scan II utilizes the principle of skiascopic phase of difference for refractive error map measurement [14]. The retina is scanned with an infrared light slit beam, and the reflected light is captured by an array of rotating photodetectors over a 360° area. By using 808 nm wavelength light, it takes 1440 data point measurements (highest number of all wavefront machines) in approximately 0.4 seconds. OPD-Scan II creates wavefront total order aberration map, wavefront higher order aberration map, and Zernike graph. OPD wavefront refraction root-mean-square (RMS) values at 3 mm (RMS-3) and 5 mm (RMS-5) were recorded simultaneously in the same recording. The OPD RMS values represent the distribution of refractive power caused by all aberrations of the eye. The higher this value is, the more likely that there is irregular astigmatism or high levels of wavefront aberration within the optical system that could reduce visual quality. Normal level of RMS-3 and RMS-5 is ≤0.5 D.

Pupil diameter measurements were performed by using the pupillometer incorporated in the NİDEK OPD-Scan II system. Three measurements were obtained in the mesopic and photopic conditions. The mean of the measurements is presented as an output. Automatic quality check function rejects the bad measurements. The pupil camera was used to capture images of each eye in a closed and darkened room with the illumination of <0.085 lx between 08:30 am and 11.00 am under two natural undilated illumination conditions (mesopic: 10 lx, photopic: 100 lx). All OPD measurements were performed after 15 min of dark adaptation for each eye.

## 3. Data and Statistical Analysis

### 3.1. Vector Analysis

Vector analysis proposed by Thibos and Horner was used to convert spherocylindrical refractive errors (*S* [sphere], *C* [cylinder] *xφ* [axis]) into a set of three dioptric powers: *M*, *J*_0_, and *J*_45_, by the following formulas [15]:

(1) $$\begin{array}{c} M=S+\frac{C}{2};\;\;J_{0}=\left(-\frac{C}{2}\right)\cos\left(2\varphi \right);\\ J_{45}=\left(-\frac{C}{2}\right)\sin\left(2\varphi \right);\\ B={\left(M^{2}+{J_{o}}^{2}+{J_{45}}^{2}\right)}^{1/2}, \end{array}$$

where *M* denotes the spherical equivalent refraction, *B* denotes the overall blur strength of the refractive error, *C* denotes mean cylinder, *J*_45_ denotes the power of Jackson cross cylinder at axis 45°, and *J*_0_ denotes the power of the Jackson cross cylinder at axis 180°. This mathematical approach has the advantage that astigmatism is represented in rectangular vector form, allowing statistical analysis to be applied to each component separately (orthogonality) and test hypotheses [15, 16].

### 3.2. Analysis of Wavefront Aberrations

Under standardized light conditions, three consecutive measurements were taken with the OPD-scan wavefront analyzer. Mean values were obtained for statistical analyses. The analyzed parameters included

1. RMS of higher order aberrations (HOA) from the third to eighth orders;
2. RMS of the total spherical aberration (TSA) (square root of the sum of the squared coefficients of *C* ^0^_4_, *C* ^0^_6_, and *C* ^0^_8_);
3. RMS of total coma (TC) (square root of the sum of the squared coefficients of *C* ^−1^_3_, *C* ^1^_3_, *C* ^−1^_5_, *C* ^1^_5_, *C* ^−1^_7_, and *C* ^1^_7_);
4. RMS of total trefoil (T3F) (square root of the sum of the squared coefficients of *C* ^−3^_3_, *C* ^3^_3_, *C* ^−3^_5_, *C* ^3^_5_, *C* ^−3^_7_, and *C* ^3^_7_);
5. RMS of total tetrafoil (T4F) (square root of the sum of the squared coefficients of *C* ^−4^_4_, *C* ^4^_4_, *C* ^−4^_6_, *C* ^4^_6_, *C* ^−4^_8_, and *C* ^4^_8_);
6. RMS of total high order astigmatism (HiA) (square root of the sum of the squared coefficients of *C* ^−2^_4_, *C* ^2^_4_, *C* ^−2^_6_, *C* ^2^_6_, *C* ^−2^_8_, and *C* ^2^_8_).

The difference between presmoking and postsmoking value of each parameter was defined as

(2) $$\begin{array}{c} \Delta \text{data}={\text{data}}_{\text{postsmoking}}-{\text{data}}_{\text{presmoking}}. \end{array}$$

### 3.3. Statistical Analysis

SPSS version 11.0 statistical software system was used for all statistical analyses and *P* < 0.05 was regarded as statistically significant. Only the right eyes were recruited for statistical analysis. The differences of pupil sizes and ocular aberrations before smoking and immediately after smoking were analyzed by Wilcoxon signed ranks test.

## 4. Results

Fifty-two eyes of 26 male participants with a mean age of 34.5 ± 4.6 years were evaluated. Mean spherical power was −0.52 ± 0.67 diopters and mean cylindrical power was 0.31 ± 0.52 diopters. Mean photopic pupil size was 3.52 ± 0.73 mm before smoking. Photopic pupil size was found to decline to 3.29 ± 0.58 mm immediately after smoking (*P* = 0.001). Mean mesopic pupil size also decreased from 6.42 ± 0.75 mm to 6.14 ± 0.75 mm after smoking (*P* = 0.001).

The mean RMS-3 and RMS-5 were 0.23 ± 0.16 D and 0.61 ± 1.03 before smoking which decreased to 0.21 ± 0.13 D and 0.39 ± 0.43 D after smoking. However, there were no statistically significant differences in mean RMS-3 and RMS-5 parameters after smoking (*P* = 0.703 and *P* = 0.770, resp.).

Mean pre-/postsmoking higher order aberrations and Zernike terms are demonstrated in Table 1. Although there was a decrease in mean values of TWA, HOA, TC, T3F, T4F, TSA, and HiA, the changes in ocular aberrations after smoking were found to be statistically indifferent from the presmoking values.

## 5. Discussion

Widespread tobacco addiction is mainly caused by nicotine which is a tertiary amine composed of a pyridine and a pyrrolidine ring. The effects of nicotine are initiated by binding to nicotinic cholinergic receptors in autonomic ganglia, adrenal medulla, and neuromuscular junctions as well as in the central nervous system [1]. Binding of nicotine to cholinergic receptors causes the release of a number of vasoactive catecholamines and neuroactive peptides [17, 18]. Being the only active pharmacological agent in tobacco, nicotine increases respiration rate, heart rate, blood pressure, and coronary blood flow outside central nervous system and increases cognitive functioning, psychomotor activity, and sensorimotor performance in the central nervous system.

Cigarette smoking has been found to be related to cataract formation [19], age-related macular degeneration [20], retinal vein occlusion [21], anterior ischemic optic neuropathy [22], and thyroid ophthalmopathy [23]. The sphincter muscle of iris contracts resulting in miosis after cholinergic impulse, whereas the radial muscle of iris contracts resulting in mydriasis after *α*1 noradrenergic receptor impulse. To the best of our knowledge, effects of tobacco smoking on pupil size were not clearly established. Lie and Domino investigated pupil diameter, heart rate, and systolic and diastolic blood pressure before and after sham or tobacco smoking in 20 healthy adults [24]. They measured pupil size from a colored photograph taken with a polaroid camera. Although both nonsmokers and smokers showed slight but statistically significant pupillary constriction after sham or tobacco-smoking, miosis was greater after tobacco-smoking.

In the present study, both photopic and mesopic pupil diameters were measured using an automated pupillometer rather than standard anterior segment photography. Our results revealed a significant decrease both in photopic and mesopic pupil sizes after tobacco smoking. A mean decrease of 0.23 ± 0.39 mm in photopic pupil size and a mean decrease of 0.26 ± 0.24 mm in mesopic pupil size were recorded. It appears that the sphincter of the iris is relatively more activated by the parasympathetic nervous system than sympathetic system. This finding is surprising because in the cardiovascular system tobacco smoking activates predominantly the sympathetic nervous system.

In addition, psychologic relaxation after smoking in chronic smokers [25] may also cause the inhibition of sympathetic activation in the abstinence period. The chronic smokers in this study had an abstinence period for at least 12 hours before the measurements. On this basis, further studies are warranted to explore the effect of smoking on pupil size and ocular aberration with and without abstinence from smoking. Although photopic and mesopic pupil size differences between presmoking and postsmoking sessions were statistically significant, it does not absolutely mean that the differences have clinical significance. Mean pupil size changes were only 0.23 mm and 0.26 mm in photopic and mesopic measurements, respectively.

Pulmonary absorption of nicotine is extremely rapid, occurring at a similar rate of intravenous administration. Cotinine is the primary metabolite of nicotine. Cotinine levels tend to increase gradually during the day, peaking at the end of smoking and persisting in high concentrations overnight [26]. Therefore, in the current study, the participants were asked to abstain from tobacco smoking for a night.

Wavefront aberrometry is a method used for the objective assessment of visual performance and calculates higher order ocular aberrations. In clinical practice, evaluation of higher order aberrations which affect retinal image quality is mostly used in keratorefractive surgery [27]. Among higher order aberrations, coma-like aberration was reported to be primarily responsible for the loss of contrast sensitivity in healthy human eyes [28]. Not only spherical and coma aberrations but also higher order aberrations contribute considerably to reduction in visual quality when the pupil is larger [12, 29–34]. At 3 mm pupil, small number of sensor elements are involved in the wavefront inclination measurement [9]. Although we detected a significant miosis in both photopic and mesopic measurements, wavefront aberrations of the eye did not seem to alter significantly after smoking despite a slight decrease.

The accommodation status is also known to be of relevance during wavefront sensing [27]. One weakness of our study is that the accommodation of the patients was not evaluated. Since accommodation is achieved by the changes in the shape and position of the lens, wavefront aberrations are supposed to change with accommodation [35]. Spherical aberration was shown to be more negative with the increase of accommodation effort. Also accommodation was found to produce significant changes in coma [29].

The influence of tobacco smoking on pupil diameter and ocular higher order aberrations have not been reported previously. In view of the aforementioned results, our results revealed in the acute period that tobacco smoking causes dominantly miosis when pupil diameter is considered. Therefore, as a result, we may conclude that the sphincter of the iris is relatively more activated by the parasympathetic nervous system. Unexpectedly, pupillary constriction observed after tobacco smoking did not decrease wavefront aberrations notably.

## Figures and Tables

**Table 1:** Pre- and postsmoking higher order aberrations detected by OPD-Scan II.

	RMS-3			RMS-5		
	Before smoking	After smoking	*P* ^*^	Before smoking	After smoking	*P* ^*^
TWA	1.37 ± 1.02	1.16 ± 0.85	0.990	1.45 ± 0.99	1.40 ± 0.75	0.134
HOA	0.62 ± 0.89	0.40 ± 0.31	0.374	0.68 ± 0.81	0.062 ± 0.36	0.345
TC	0.25 ± 0.24	0.18 ± 0.14	0.568	0.28 ± 0.20	0.22 ± 0.16	0.063
T3F	0.35 ± 0.43	0.26 ± 0.25	0.657	0.39 ± 0.27	0.34 ± 0.24	0.089
T4F	0.19 ± 0.43	0.08 ± 0.11	0.819	0.22 ± 0.38	0.18 ± 0.15	0.143
TSA	0.14 ± 0.13	0.09 ± 0.07	0.238	0.16 ± 0.10	0.14 ± 0.17	0.138
HiA	0.13 ± 0.22	0.06 ± 0.05	0.084	0.17 ± 0.20	0.15 ± 0.15	0.716

TWA = total wavefront aberrations, HOA = higher order aberration, TC = total coma, T3F = total trefoil, T4F = total tetrafoil, TSA = total spherical aberration, and HiA = total higher order astigmatism.

^*^Wilcoxon signed ranks test.
